# Pharmacokinetics and Scintigraphic Imaging of the Hypoxia-Imaging Agent [^123^I]IAZA in Healthy Adults Following Exercise-Based Cardiac Stress †

**DOI:** 10.3390/pharmaceutics10010025

**Published:** 2018-02-22

**Authors:** Daria Stypinski, Stephen A. McQuarrie, Alexander J. B. McEwan, Leonard I. Wiebe

**Affiliations:** 1 Clinical Pharmacokinetics, Pfizer Inc., New York, NY 10017, USA; Daria.Stypinski@pfizer.com; 2 PET Centre, Department of Oncology, University of Alberta, 11560 University Ave, Edmonton, AB T6B 1Z2, Canada; smcquarr@ualberta.ca (S.A.M.); sandy.mcewan@albertahealthservices.ca (A.J.B.M.); 3 Faculty of Pharmacy and Pharmaceutical Sciences, and Department of Oncology, University of Alberta, Edmonton T6G 2R3, Canada; 2-40 Medical Isotope & Cyclotron Facility, University of Alberta-South Campus, Edmonton, AB T6H 2V8, Canada

**Keywords:** pharmacokinetics, radiotracers, hypoxia, nuclear imaging, [^123^I]IAZA

## Abstract

The objective of this work is to evaluate the potential effect of cardiac stress exercise on the accumulation of [^123^I]IAZA, a radiopharmaceutical used to image focal tissue hypoxia, in otherwise normal myocardium in healthy volunteers, and to determine the impact of exercise on [^123^I]IAZA pharmacokinetics. The underlying goal is to establish a rational basis and a baseline for studies of focal myocardial hypoxia in cardiac patients using [^123^I]IAZA. Three healthy male volunteers ran the ‘Bruce’ treadmill protocol, a clinically-accepted protocol designed to expose myocardial ischemia in patients. The ‘Bruce’ criterion heart rate is 85% of [220–age]. Approximately one minute before reaching this level, [^123^I]IAZA (5.0 mCi/0.85 mg) was administered as a slow (1–3 min) single intravenous (i.v.) injection via an indwelling venous catheter. The volunteer continued running for an additional 1 min before being transferred to a gamma camera. Serum samples were collected from the arm contralateral to the administration site at pre-determined intervals from 1 min to 45 h post injection and were analyzed by radio HPLC. Pharmacokinetic (PK) parameters were derived for [^123^I]IAZA and total radioactivity (total[^123^I]) using compartmental and noncompartmental analyses. Whole-body planar scintigraphic images were acquired from 0.75 to 24 h after dosing. PK data and scintigraphic images were compared to previously published [^123^I]IAZA data from healthy volunteers rest. Following exercise stress, both [^123^I]IAZA and total[^123^I] exhibited bi-exponential decline profiles, with rapid distribution phases [half-lives (t_1/2α_) of 1.2 and 1.4 min, respectively], followed by slower elimination phases [t_1/2β_ of 195 and 290 min, respectively]. Total body clearance (CL_TB_) and the steady state volume of distribution (V_ss_) were 0.647 L/kg and 185 mL/min, respectively, for [^123^I]IAZA and 0.785 L/kg and 135 mL/min, respectively, for total[^123^I]. The t_1/2β_, CL_TB_ and V_ss_ values were comparable to those reported previously for rested volunteers. The t_1/2α_ was approximately 4-fold shorter for [^123^I]IAZA and approximately 3-fold shorter for total[^123^I] under exercise relative to rested subjects. The heart region was visualized in early whole body scintigraphic images, but later images showed no accumulated radioactivity in this region, and no differences from images reported for rested volunteers were apparent. Minimal uptake of radiotracer in myocardium and skeletal muscle was consistent with uptake in non-stressed myocardium. Whole-body scintigrams for [^123^I]IAZA in exercise-stressed healthy volunteers were indistinguishable from images of non-exercised volunteers. There was no evidence of hypoxia-dependent binding in exercised but otherwise healthy myocardium, supporting the conclusion that exercise stress at Bruce protocol intensity does not induce measurable myocardial hypoxia. Effects of exercise on PK parameters were minimal; specifically, the t_1/2α_ was shortened, reflecting increased cardiac output associated with exercise. It is concluded that because [^123^I]IAZA was not metabolically bound in exercise-stressed myocardium, a stress test will not create elevated myocardial background that would mask regions of myocardial perfusion deficiency. [^123^I]IAZA would therefore be suitable for the detection of viable, hypoxic myocardium in patients undergoing stress-test-based diagnosis.

## 1. Introduction

**Figure 1:**
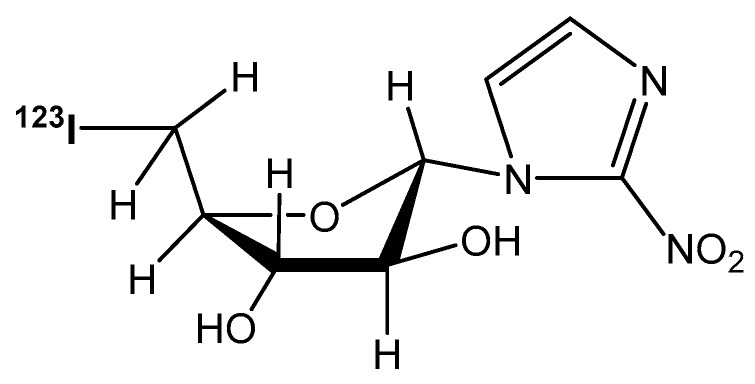
Chemical structure of 1-α-d-(5-deoxy-5-[^123^I]iodoarabinofuranosyl)-2-nitroimidazole ([^123^I]iodoazomycin arabinoside; [^123^I]IAZA).

Nuclear cardiology assesses myocardial viability and establishes a prognosis for cardiac patients [1,2,3], using a range of radiotracers to observe myocardial perfusion and bioenergetics [4,5]. An alternative imaging approach is to detect pathological, oxygen deficient (hypoxic) myocardial tissue using oxygen-sensitive radiotracers, which offers the advantage of providing prognostic data on ischemic but salvageable myocardium. Radiolabeled azomycins (2-nitroimidazoles such as [^18^F]FMISO, [^18^F]FAZA, [^123^I]IAZA, [^99m^Tc]BMS-181321) and others have long been shown to accumulate selectively in hypoxic tissues [6,7], including ischaemic myocardium [8]. In theory, these radiotracers are metabolically trapped and will concentrate in hypoxic myocardium that has intracellular oxygen partial pressures below about 3 mm Hg (<25% of normal) and would therefore be useful for assessing myocardial damage. [^123^I]Iodoazomycin arabinoside ([^123^I]IAZA (Figure 1) was developed as an imaging agent for the detection of hypoxic, radiation-resistant regions in solid tumors in cancer patients [9,10,11,12]. It is efficient as a marker of clinical hypoxia in peripheral vascular disease of diabetic origin [13], blunt brain trauma [14], and rheumatoid joints [15]. The associated clinical pharmacokinetics (PK) and radiation dosimetry in healthy volunteers [16,17] have been previously published [18].

There is now renewed interest in determining the suitability of [^123^I]IAZA as a tool in assessment of myocardial viability in cardiac patients, which requires, as first steps, evaluation of: (1) the impact of physical stress on baseline chest cavity images and (2) any changes to [^123^I]IAZA and total radioactivity PK that may adversely affect target uptake and background clearance. Specifically, it is necessary to ensure that exercise stress does not induce a general increase in myocardial tissue background to the extent that a hypoxic lesion would be masked during a stress test. This paper presents [^123^I]IAZA PK data and planar scintigraphic images for three healthy male volunteers, each of whom received a single i.v. bolus dose of [^123^I]IAZA while performing strenuous cardiac exercise. These data are compared with previously published results [16,17] in six healthy, rested (no exercise) subjects who were administered similar [^123^I]IAZA doses.

## 2. Materials and Methods

All clinical procedures were conducted following the tenets of the Declaration of Helsinki (1964) and were approved by the Alberta Cancer Board Research Ethics Committee (ETH-95-52-19).

### 2.1. Blood and Urine Sample Analysis and Dosimetry after Intravenous Administration of ^123^I-IAZA to Volunteers

Three healthy male volunteers (Table 1), aged 27 to 42 years, weighing 70 to 75 kg, participated following informed consent. Lugol's solution (0.6 mL, USP) in orange juice was given orally to block uptake of radioiodide by the thyroid, and shortly thereafter, each volunteer ran the ‘Bruce’ treadmill protocol. In this test, the criterion heart rate was 85% of [220–age] [19]. Approximately one minute before reaching the target heart rate, each subject received approximately 5.0 mCi/0.85 mg [^123^I]IAZA (nominally 185 MBq) as a slow (1–3 min) i.v. bolus injection into a pre-cannulated arm vein. The volunteer then continued running until the injection was completed, about an additional 1 min.

### 2.2. Pharmacokinetic Analysis (PK)

Fourteen venous blood samples (9 mL each) were collected into SST Vacutainer^®^ tubes at pre-determined intervals from an indwelling catheter (positioned in the arm contralateral to the dosing arm) for analysis of serum [^123^I]IAZA and total radioactivity (total[^123^I]), beginning at pre-dose (0 h) and then post-dose from 1 min to 45 h. Serum samples were analyzed using previously reported serial radiometric high performance liquid chromatography (radioHPLC) [20]. Concentrations of [^123^I]IAZA and total[^123^I] in serum were analyzed via compartmental and noncompartmental (NCA) methods using WinNonlin version 1.1 (Scientific Consulting Inc., Apex, NC, USA, 2002). For compartmental analysis, the choice of model was based on observed and calculated correlation function values, Akaike (AIC) and Schwartz (SC) criteria, and linearity of the partial derivatives plots, with the iteratively reweighted least squares weighing option. Since ^123^I has a 13.2 h physical decay half-life, instrumental results were decay-corrected for the difference in time from radiometry to the time when the sample was collected.

There is no reported evidence of dose-dependency in [^123^I]IAZA PK and hypoxia imaging in the 0.1 mg to 10 mg dose range [16,18]. For optimal image quality, the primary target at the time of administration was the nominal radioactive dose. The relatively short physical decay half-life of ^123^I resulted in rapidly declining specific activity (i.e. MBq/mg) of the dosing preparations at the time of injection. This unavoidably results in considerable variability in the individual mass doses of IAZA, including those administered to subjects in the current study (0.57 mg to 1.18 mg, mean of 0.85 mg) and the reference study in rested volunteers (Table 1). For this reason, the primary PK parameters of interest for comparison between the two studies were limited to dose-independent parameters, including the distribution half-life (t_1/2α_) and elimination half-life (t_1/2β_), as determined using compartmental methods, and steady-state volume of distribution (V_ss_) and total body clearance (CL_TB_), determined using NCA.

### 2.3. Imaging

Three SPECT scintigraphs of the chest cavity and five anterior and posterior whole body planar images were acquired at different time periods post injection, beginning immediately after completion of the treadmill protocol (approximately 0.75 h), and at 1–2, 3–4, 6–8 and 20–24 h post injection. All image acquisition times were 30 min, using a dual-headed, large field of view gamma camera (Picker Odyssey 2000, Picker International Canada Inc., Brampton, ON, Canada) equipped with a LEAP (low-energy all-purpose) collimator and an Odyssey computer. A 20% analysis window was set symmetrically over the 159 keV I-123 photopeak.

## 3. Results

All three subjects who were enrolled in the study completed the protocol with no apparent adverse events. Demographic and dosing information are presented in Table 1.

### 3.1. Pharmacokinetics

**Figure 2:**
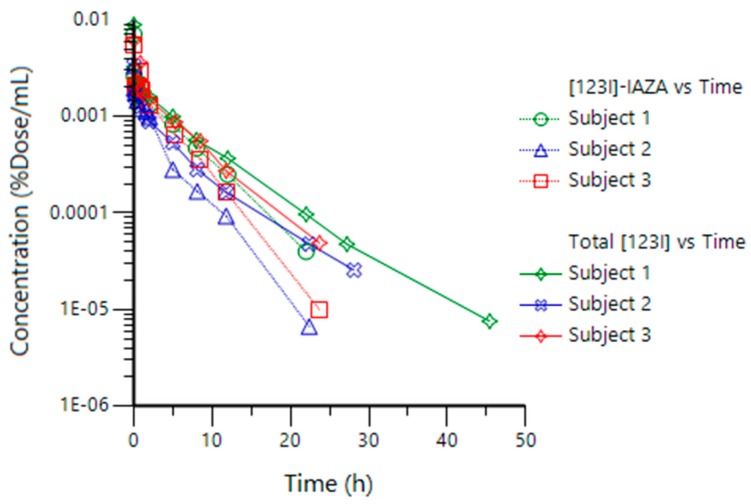
Concentration-time plots for [^123^I]IAZA and total[^123^I] in each subject.

The %Dose concentration vs time plots for [^123^I]IAZA and for total[^123^I] for the three volunteers undergoing the “Bruce” treadmill protocol are shown in Figure 2.

Both [^123^I]IAZA and total[^123^I] showed bi-exponential decline profiles following an i.v. bolus dose, with a fast distribution phase (t_1/2α_ of 1.2 ± 0.15 and 1.4 ± 0.28 min, respectively), followed by longer elimination phase (195 ± 34 and 290 ± 37 min, respectively). A 2-compartment open model with an i.v. bolus input function provided the best fit for the PK data for both [^123^I]IAZA and total[^123^I] in all subjects; this was also the case with the group of rested volunteers [16]. The individual and mean (± SD) compartmental (t_1/2α_, t_1/2β_), and non-compartmental (V_ss_ and CL_TB_) PK parameters of interest from the cardiac-stressed volunteers in this study are presented in Table 2, along with comparative historical data for the 6 healthy volunteers dosed under resting conditions [16].

### 3.2. Imaging

**Figure 3:**
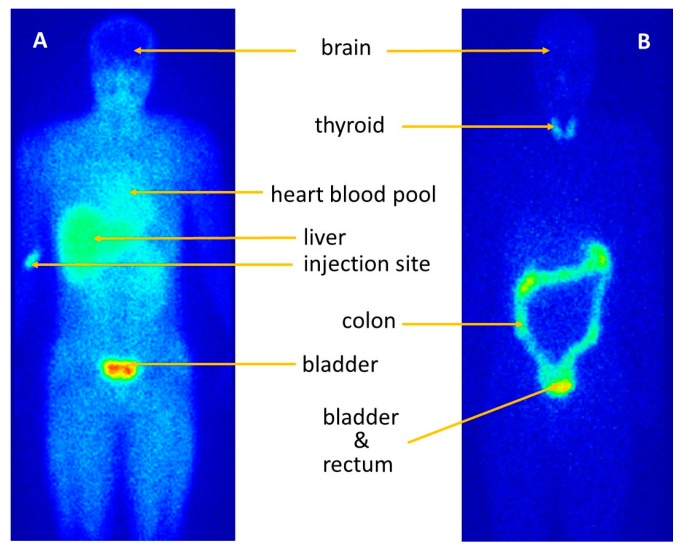
Planar anterior early (**A**) and late (**B**) images depicting radioactivity distribution following i.v. injection of [^123^I]IAZA in an exercising volunteer. The left image (**A**), acquired 15–45 min after injection, shows extensive distribution of radioactivity throughout soft tissues, including viscera, skeletal muscle and heart/blood pool, but not brain. The right image (**B**), acquired 22 h after injection, shows that radioactivity has effectively been cleared from the body except for thyroid (estimated at 0.5% of the dose) and large bowel (estimated at 5% of the dose). Images reflect identical total counts/image, which necessitated longer imaging time for the 22 h image because by then most of the radioactivity had decayed (~2 × physical T_1/2_) and/or been excreted.

The myocardium and skeletal muscles were only visible in the initial whole body and SPECT images, but not on the later images in this study (Figure 3). The early, intermediate and late post-dosing whole-body images were consistent with those reported for the rested volunteers [17]. On the earliest images, the organs with the highest radioactivity accumulation were the bladder, liver and the kidneys. The i.v. injection site was also visible on the immediate images of one volunteer, but the percent of radioactive dose in that area, as estimated by image inspection, was negligible and therefore not expected to alter the distribution kinetics of the radiopharmaceutical. There was a striking absence of radioactivity in the brains of all the volunteers in early images and in intermediate-time images, indicating effective exclusion of [^123^I]IAZA by the blood brain barrier. Later images, however, showed some redistribution of radioactivity into the brain. Immediate, 1–2 h and 3–4 h images also did not show thyroid or large intestine uptake, but these organs were visible at late (20–24 h) image acquisition times.

## 4. Discussion

Diagnostic imaging techniques depend on good image contrast for effective interpretation. In nuclear cardiology, this is especially so because the heart-chamber blood pool and the myocardium blood perfusion volume can significantly elevate the background. The kinetics of clearance of the radioactive diagnostic agent from the blood therefore governs the rate at which a suitable contrast will arise. Slow clearance ‘competes’ with a short radiotracer effective half-life, an effect that is compounded by a short radioisotope physical decay half-life and by the background clearance (usually blood) half-life. In practical terms, slow clearance from the blood implies delayed imaging, which in turn has both logistical and economic consequences. A further challenge relates to rates of metabolic uptake and clearance of the radiotracer by the hypoxic region in question. In order for [^123^I]IAZA to be an effective diagnostic of myocardial lesions (focal ischemia), it is necessary to know when the ratios of background to lesion concentrations provide optimal contrast under the conditions of the test. Because the stress test is an important component of nuclear cardiology imaging, it is essential to know how the radiotracer will behave under exercise stress conditions: (how) will exercise-induced cardiac output affect radiotracer kinetics, and will the myocardium itself develop sufficient hypoxia to induce bioreductive binding of the tracer, thereby creating a high, long-lived background that could mask uptake by the lesion.

The current work shows that acute strenuous exercise appears to have little impact on the PK of either [^123^I]IAZA or total[^123^I] after doses of [^123^I]IAZA. Only t_1/2α_ was shortened in subjects undergoing the “Bruce” treadmill protocol (1.2 min) relative to rested subjects (5.3 min) [16], which was not unexpected, given that strenuous exercise increases both cardiac output and tissue perfusion, hastening drug distribution for lipophilic drugs such as [^123^I]IAZA. Other PK parameters; t_1/2β_, V_ss_ and CL_TB_, for both [^123^I]IAZA and total[^123^I], were comparable between stressed subjects and reference subjects dosed at rest. Since [^123^I]IAZA distribution is rapid and of short duration, it contributes only approximately 5% of the overall exposure in subjects at rest [16], and therefore any change in the distribution rate would be expected to have negligible impact on the overall PK profiles of both [^123^I]IAZA and total radioactivity. Given the 30-min image acquisition time, the distribution phase was too short to be discernable with gamma camera scintigraphic techniques, a limitation imposed by gamma camera sensitivity and the amount of [^123^I]IAZA injected. Since the difference in distribution phase could not be captured, the whole-body images obtained from exercising volunteers were deemed consistent with those reported for rested volunteers [17].

The myocardium and skeletal muscles were visible on the initial whole-body and SPECT images of subjects undergoing exercise. However, all whole-body images (early, intermediate and late) were consistent with whole-body images in rested subjects [17]. These results indicate that myocardial radioactivity observed in the early images (Figure 3A) is most likely attributable to blood pool radioactivity in the heart chambers and in highly perfused cardiac muscle, and not to any stress-induced hypoxia-related retention. This absence of active stress-induced uptake indicates that [^123^I]IAZA may be suitable for the detection of viable, hypoxic myocardium in areas of myocardial perfusion deficiency. In clinical practice, an imaging procedure could be performed at around 8 h after dosing, giving sufficient time for background radiation associated with blood pool to be adequately cleared so that any metabolically bound [^123^I]IAZA in the hypoxic myocardium would be clearly distinguishable in the scans.

Accumulation of radioactivity by the large intestine is visible on late (20–24 h post-dose) images. This may be indicative of a minor route of elimination via biliary excretion of radioactivity into the gut, which was estimated to represent only approximately 5% of the administered dose. This small amount constitutes a significant relative contribution to the whole-body radioactivity in the late image, given that, as determined from renal excretion of total radioactivity in the six healthy volunteers dosed at rest on the prior study [17], 92% of decay-adjusted total radioactivity was eliminated renally within 28 h post-dose. Since only about 15% of that radioactivity was attributed to intact [^123^I]IAZA, the remaining 85% was from radiolabeled metabolites, including free [^123^I]iodide originating from metabolic deiodination of [^123^I]IAZA. It is possible that some of these metabolites may be excreted into the bile, to be eliminated in feces.

The volunteers in this study received a single oral dose of Lugol’s solution immediately before [^123^I]IAZA injection, to block uptake of [^123^I]iodide by the thyroid [21]. Quality control during manufacture of the dose assured the absence of free [^123^I]iodide in the injection. However, the thyroid gland, which was barely visible in early images, was clearly present in the late (20–24 h) images. Accumulation of radioactivity in the thyroid gland is taken as evidence of metabolic deiodination. In the case of [^123^I]IAZA, accumulated [^123^I]iodide represented approximately 0.5% of administered dose. It was concluded that a single dose of cold iodide (Lugol’s solution) did not provide a full, lasting blockage, an observation consistent with other reports in the literature [22]. For the exercise study, the radioactivity in the thyroid gland was considered irreversibly bound and was therefore eliminated with the 13.2 h physical half-life of the isotope. Since iodine incorporation into the thyroid gland takes place with a 6–8 h half-life, it may be possible to further decrease the radiation dose to the thyroid by administering Lugol’s solution earlier relative to the [^123^I]IAZA dose [21,22].

The concentration-time plot of total radioactivity following [^123^I]IAZA administration exhibited a bi-exponential decline (Figure 1), and therefore only two compartments were discernible. However, gamma camera scintigraphic images of the whole body delineate several physiological regions with elevated radioactivity, including liver, kidney and thyroid. Radiopharmaceutical imaging is, therefore, a natural element of physiologically based pharmacokinetic (PBPK) modelling. PBPK can be used to assess the exposure in a target organ after dosing, taking into account organ-specific absorption, metabolism and disposition rates in that organ [23]; it does not rely heavily on plasma or serum PK to elucidate all of the physiological compartments. In the current imaging work, attempts to discern whether these regions were indeed separate compartments of a multicompartmental pharmacokinetic model for [^123^I]IAZA proved unsuccessful. This was attributed to the facts that the scintigraphic images reflect total radioactivity and that [^123^I]IAZA is known to be extensively metabolized. For example, the thyroid represents less than 0.5% of the all cumulative body activity, but because all of it is attributable to free [^123^I]iodide, it would not constitute a distinct compartment in a physiologically based PK model for [^123^I]IAZA. The critical limitation to the use of imaging techniques to aid PBPK modeling is that scintigraphic imaging ‘sees’ all radioactivity regardless of its chemical form, i.e., [^123^I]IAZA plus all its radiolabeled metabolites.

## 5. Conclusions

Whole-body scintigrams for [^123^I]IAZA in exercise-stressed healthy volunteers were indistinguishable from images of non-exercised volunteers. There was no evidence of hypoxia-dependent binding in exercised but otherwise healthy myocardium, supporting the conclusion that exercise stress at Bruce protocol intensity does not induce measurable myocardial hypoxia. Effects of exercise on PK parameters were minimal; specifically, the t_1/2α_ was shortened, reflecting increased cardiac output associated with exercise. It is concluded that because [^123^I]IAZA was not metabolically bound in exercise-stressed myocardium, it would not create an elevated background that would mask regions of myocardial perfusion deficiency, and would therefore be suitable for the detection of viable, hypoxic myocardium in patients undergoing stress-test-based diagnosis.

Further studies in patients with fully characterized, focal myocardial ischemia are now warranted.

## Figures and Tables

**Table 1:** Exercise-stressed healthy volunteer demographics and individual IAZA doses. Each subject received a nominal 5 mCi injection of [^123^I]IAZA. Published data for rested volunteers [16] are included for comparison.

Subject number	Sex	Age (y)	Weight (kg)	Height (cm)	IAZA Dose (mg)
V1	M	42	70	175	0.80
V2	M	27	75	186	1.18
V3	M	40	73	165	0.57
Mean ± SD	-	36 ± 8	73 ± 3	175 ± 11	0.85 ± 0.31
Reference Study [16] *	4 M 2 F	37 ± 13	80 ± 10	175 ± 9	0.68 ± 0.44

* Mean ± SD, N = 6.

**Table 2:** Pharmacokinetic (PK) parameters for [^123^I]IAZA and total[^123^I] in exercise-stressed volunteers. Published data for rested volunteers [16] are included for comparison.

	PK	[^123^I]IAZA				total[^123^I]			
Subject		t_1/2α_ (min)	t_1/2β_ (min)	V_ss_ (L/kg)	CL_TB_ (mL/min)	t_1/2α_ (min)	t_1/2β_ (min)	V_ss_ (L/kg)	CL_TB_ (mL/min)
V1		1.1	234	0.677	145	1.3	328	0.794	104
V2		1.3	170	0.803	254	1.2	287	0.979	182
V3		1.1	182	0.541	157	1.8	254	0.581	120
Exercise Mean ± SD		1.2 ± 0.1	195 ± 3	0.647 ± 0.1	185 ± 60	1.4 ± 0.3	290 ± 4	0.785 ± 0.2	135 ± 4
Rested [16] *		5.3 ± 3.8	179 ± 3	0.716 ± 0.1	239 ± 5	4.6 ± 2.6	294 ± 3	0.746 ± 0.1	145 ± 19

* Mean ± SD, N = 6.
